# Excessive smartphone use in Tibet Autonomous Region: biopsychosocial and health behavior factors

**DOI:** 10.3389/fpsyt.2025.1645243

**Published:** 2025-10-07

**Authors:** Ruixue Zhao, Yongzhulacuo, Zhaoyu Li, Stephen Nicholas, Elizabeth Maitland, Zhaxidawa, Wenhua Wang

**Affiliations:** ^1^ School of Public Policy and Administration, Xi’an Jiaotong University, Xi’an, Shaanxi, China; ^2^ Medical College, Xizang University, Lhasa, China; ^3^ Key Laboratory of Public Health Safety &Policy Research, Xizang University, Lhasa, China; ^4^ School of Pharmaceutical Science, Wenzhou Medical University, Wenzhou, Zhejiang, China; ^5^ Health Services Research and Workforce Innovation Centre, Newcastle Business School, The University of Newcastle, Newcastle, NSW, Australia; ^6^ Australian National Institute of Management and Commerce, Sydney, NSW, Australia; ^7^ School of Management, University of Liverpool, Liverpool, United Kingdom

**Keywords:** excessive smartphone use, addiction, biopsychosocial model, mental health, depression

## Abstract

**Backgrounds:**

Excessive smartphone use is a growing global health concern, with most research focusing on adolescents. Across all age groups, we assess the impact of regional, biopsychosocial and health behavior factors on excessive smartphone use in a unique ethnic group, Tibetans, in China.

**Methods:**

Through systematic random sampling, we conducted a cross-sectional survey using the Mobile Phone Smartphone use Questionnaire (MPIQ) among 1,374 Tibetan residents from Shannan, Nyingchi, and Qamdo regions (October-December 2023). Data analysis included descriptive statistics, chi-square tests, one-way ANOVA, and multinomial logistic regression within a biopsychosocial framework.

**Results:**

Among the 1374 respondents, 30.28% were excessive smartphone users, with high excessive use rates across all age groups, including 20–39 young adults (40.53%), 40–65 middle-aged (26.86%) and over 65 (21.23%) aged groups. Significant regional disparities showed higher use in Qamdo and Nyingchi than in Shannan region. Males and individuals with higher depression scores (measured by the PHQ-9) more prone to excessive smartphone use, while regular physical exercise emerged as a protective factor. Socioeconomic factors, including education level, income level and water source, played a significant role in excessive smartphone use.

**Conclusion:**

With nearly one-third of the Tibetan sample excessively engaged in smartphone use, targeted and multifaceted public health interventions are essential. Interventions should holistically address both mental and physical health by integrating depression support and vigorously promoting physical activity. Furthermore, strategies must be tailored for key demographics, recognizing the risks facing young and middle-aged adults, men, and high-socioeconomic-status individuals. Finally, to ensure cultural resonance and effectiveness, interventions should leverage Tibet’s strong community ties by promoting traditional face-to-face interactions and be adapted to significant regional differences.

## 1 Introduction

### 1.1 Background

The global proliferation of online services—from remote work and e-commerce to digital education and social interaction—has precipitated widespread digital dependence, with excessive internet and smartphone use emerging as a pressing public health concern (1). China exemplifies this trend, boasting 1.1 billion internet users as of 2024, 99.7% of whom access the web via smartphones—the highest penetration rate worldwide (CNNIC). (2, 3). This ubiquitous adoption has transformed smartphones into multifunctional lifelines, serving simultaneously as communication devices, cameras, entertainment systems, navigation tools, and productivity platforms (4). However, such technological integration comes at a cost: compulsive usage patterns—variously termed “smartphone addiction,” “problematic use,” or “nomophobia” (5–8)—are increasingly linked to anxiety, depression, sleep disturbances, musculoskeletal disorders, and even traffic accidents (9–12).

China reports the world’s highest problematic smartphone use rates (scoring 36.16/60 on standardized measures) (13), yet national statistics mask profound regional variations. Nowhere is this more apparent than in Tibet, where 90% of the 3.7 million residents are ethnic Tibetans with distinct linguistic, religious, and cultural traditions (14, 15). While Tibet’s agrarian-pastoral lifestyle might suggest lower digital engagement, three countervailing forces drive unique usage patterns: Tibetan Buddhist values and collectivist social structures may modulate digital behaviors differently than Han-majority regions; In vast, sparsely populated areas, smartphones transition from conveniences to essential tools for healthcare access, weather monitoring, and livestock management; National digital inclusion initiatives have accelerated device adoption even in remote monasteries and nomadic communities.

The current literature on smartphone use in China exhibits two critical blind spots. First, studies overwhelmingly focus on adolescents, largely neglecting adult populations whose usage patterns are distinctly shaped by work-family demands (8, 16–18). Second, ethnic minority groups are conspicuously absent from this research, particularly communities like the Tibetans, whose engagement with digital technology is mediated by unique cultural factors such as strong oral traditions (19). This gap carries real-world consequences: an intervention designed for a Shanghai teenager may prove culturally incongruent and ineffective for a Tibetan herder.

To address these blind spots, our study makes two unique contributions. First, we shift the focus from the typical adolescent cohort to examine excessive smartphone use across all age groups, including middle-aged and older adults. Second, we situate our research in the culturally distinct and understudied context of the Tibetan population. By applying an integrated biopsychosocial framework, we aim to provide a comprehensive analysis of the factors driving smartphone use in this unique demographic, where traditional lifestyles and rapid technological change intersect.

### 1.2 Conceptual framework

Understanding a complex behavioral issue like excessive smartphone use requires a multifactorial approach. This study is therefore grounded in the biopsychosocial model, which posits that health and illness are the result of a dynamic interplay between biological, psychological, and social factors (20, 21). Originally proposed by Engel to provide a more holistic perspective on disease, this model has been widely adopted to explain addiction. It suggests that the development of addictive behaviors is not caused by a single factor, but rather by a combination of biological elements (e.g., age, genetics), psychological states (e.g., depression, anxiety), and social contexts (e.g., socioeconomic status, cultural norms) (22). By integrating these domains, the biopsychosocial model provides a comprehensive framework for investigating the multifaceted drivers of excessive smartphone use.

In addition to the biopsychosocial model, this study incorporates the role of healthy lifestyle behaviors. These behaviors, such as engaging in regular physical activity and abstaining from tobacco or excessive alcohol use, are crucial actions individuals can take to prevent illness and improve overall well-being (23). Research has consistently shown that a healthy lifestyle is linked to a lower risk of multiple chronic diseases and increased longevity (24). In the context of smartphone addiction, these behaviors are particularly relevant, as they can serve as protective factors against the sedentary nature and mental health risks associated with excessive screen time (25).

By applying this integrated framework, our study aims to move beyond a simplistic analysis and achieve a more nuanced understanding of excessive smartphone use in the unique context of Tibet. We will investigate how a combination of biological, psychological, and social factors, alongside individual healthy lifestyle choices, collectively shapes smartphone usage patterns and their associated health outcomes in the Tibetan population.

## 2 Methods

### 2.1 Study design and sampling

Supported by the Tibet Autonomous Region Health Commission, a cross-sectional in-person survey systematically sampled Tibetan residents in Shannan, Nyingchi, and Qamdo regions from October to December 2023. The regions were chosen for their diversity in economic development, geography, and social structures, ensuring a representative cross-Tibetan sample. A total of 1374 individuals were surveyed face-to-face. Participants were informed of the study’s purpose and provided informed consent. To ensure data quality, the questionnaire was meticulously refined, and interviewers, trained graduates from the Medical College of Tibet University, ensured accurate data collection. A computer-assisted personal interviewing system prevented missing values, further enhancing data reliability.

### 2.2 Participants and ethical considerations

Participants were included in the study if they met the following criteria: (a) they were permanent residents of the surveyed area (having lived there for six months or longer); (b) they owned and used a smartphone; (c) were aged 12 years or older; and (d) had no cognitive impairments that would prevent them from understanding the study and providing informed consent.

This study was approved by the Biomedical Ethics Committee of Xizang University (Approval No. ZDYXLL2024010), and all procedures adhered to the principles of the Declaration of Helsinki. Informed consent was obtained from all participants prior to the interview. This study was conducted in conjunction with the 7th National Health Service Survey in Tibet, a large-scale, state-led project, and followed its established ethical protocols for community-based research.

Special considerations were made for minors and participants who were illiterate. For the small number of participants under the age of 18 (n=9), a culturally appropriate consent procedure was followed. This involved obtaining verbal consent from the head of household (*huzhu*), who is recognized as the primary decision-maker within the family. The head of household was present during the consent process, and the minor’s own assent to participate was also secured. For participants who were unable to read or write, a trained interviewer read the entire consent form aloud in their local dialect. After ensuring full understanding, these participants provided a thumbprint signature, which was co-signed by an independent witness to verify that consent was voluntary and informed.

### 2.3 Measures and variables

#### 2.3.1 Dependent variables

The study’s dependent variable, excessive smartphone use, was assessed using an adapted version of the Mobile Phone Involvement Questionnaire (MPIQ) (26). The original MPIQ consists of eight items. After consultation with local Tibetan experts to ensure cultural and linguistic applicability, we selected three core items for this study.

These three items were chosen because they directly map onto the foundational components of behavioral addiction as defined by Brown (27, 28): 1. Cognitive Salience (the activity dominates one’s thoughts), measured by the item: “I often think about my smartphone when I am not using it.” 2. Behavioral Salience (the activity dominates one’s life), measured by the item: “I often use my smartphone for no particular reason.” 3. Withdrawal (negative feelings when the activity is stopped), measured by the item: “The thought of being without my smartphone makes me feel distressed.”

Each of the three items was rated on a 7-point Likert scale (1 = strongly disagree, 2 = disagree, 3 = slightly disagree, 4 = neutral, 5 = slightly agree, 6 = agree, 7 = strongly agree). For each participant, a total score was calculated by summing the scores of the three items (ranging from 3 to 21), in line with previous research that has used similar cut-off points (29, 30). This total score was used to classify respondents into three groups: ‘excessive use’ (a score of 15 or higher), ‘average use’ (a score between 7 and 14), and ‘below-average use’ (a score of 6 or below). The 3-item scale demonstrated high internal consistency in the current study, with a Cronbach’s alpha of 0.909.

#### 2.3.2 Independent variables

Biological variables comprised age, hypertension, diabetes, other chronic diseases, body mass index (BMI), health-related quality of life (HRQoL), limitation of daily activities due to health descriptors and insomnia symptoms (31). Among them, BMI was calculated as weight in kilograms divided by the square of height in meters (kg/m²). Participants were then classified into four groups based on the World Health Organization (WHO) standard criteria: underweight (BMI<18.5), normal weight (BMI 18.5–24.9), overweight (BMI 25.0–29.9), and obese (BMI ≥ 30.0) (32).

HRQoL was measured using the EQ-5D-5L descriptive system. The system comprises five dimensions (mobility, self-care, usual activities, pain/discomfort, and anxiety/depression), with each dimension rated on five levels of severity from “no problems” to “extreme problems” (31). A participant’s health state is captured as a 5-digit number, which can be converted to a single summary index score using a country-specific “value set”. For this study, we utilized the official China-specific value set from the study by Luo et al. (33), which formally validated the scale’s use for the Chinese population. This approach provides a standardized method for assessing and comparing health states. In the current study, the EQ-5D-5L scale demonstrated good internal consistency, with a Cronbach’s alpha of 0.800.

Limitation of Daily Activities Due to Health: Participants were asked to what extent health problems had limited their daily activities over the past 6 months, using the following response options: “Not limited,” “Limited, but not severe,” and “Severely limited.” Insomnia symptoms were measured by how often participants experienced symptoms of insomnia in the past two weeks, consistent with the validated Insomnia Severity Index timeframe (34, 35). The frequency of insomnia symptoms was assessed using the following response options: “Not at all,” “Several days,” “More than half the days,” and “Almost every day.”

Psychological Factors: we measured psychological factors using depression and anxiety scales. We hypothesized that higher depression levels might lead individuals to use smartphones more frequently as a coping mechanism to escape from negative emotions or to seek social support, increasing the risk of excessive use (36). Anxiety can drive individuals to seek constant reassurance, which can result in excessive smartphone use (36).

To assess depression, we used the Chinese version of the Patient Health Questionnaire-9 (PHQ-9), which scores symptoms over the past two weeks, with a total score of 0-27, where 10 or higher indicates depression symptoms (37, 38). In the general Chinese population, the Chinese version of the PHQ-9 is a valid and efficient tool for screening depression (39). In the current study, the PHQ-9 scale demonstrated high internal consistency (Cronbach’s α = 0.779).

For anxiety, we employed the Chinese version of the Generalized Anxiety Disorder 2-item (GAD-2) scale, which has acceptable properties for identifying GAD at a cutoff score of 3 (40, 41). GAD-2 has been validated in Chinese populations and effectively detects various anxiety disorders (40, 42). In the current study, the GAD-2 scale demonstrated high internal consistency (Cronbach’s α = 0.783).

Socioeconomic Factors: we selected gender, urban-rural location, region, educational level, marital status, employment status, and total household income to reflect the diversity in social roles, expectations, and economic conditions impacting smartphone usage behavior. Additionally, we included main type of household drinking water as a proxy indicator for infrastructure development and living conditions, as different water access types reflect varying levels of infrastructure development and modernization (43), which may influence technology adoption patterns in Tibet.

Healthy Lifestyle Behavior Factors: In addition to the biopsychosocial model, we incorporated lifestyle and health behavior factors—including smoking, alcohol consumption, proactive health check-ups (excluding illness-related examinations), and weekly physical exercise (such as morning exercises, square dancing, walking, and running). Smoking status was categorized based on participant self-report into ‘smokers’ (defined as those currently smoking at least one cigarette per day) and ‘non-smokers/ex-smokers’. These behavioral variables offer a comprehensive perspective on health-related lifestyle behaviors that may influence smartphone use, capturing dimensions beyond biological, psychological, and socioeconomic factors.

### 2.4 Statistical analysis

Cronbach’s alpha assessed scale reliability. Descriptive statistics were reported for all variables, and univariate analysis using χ² tests and One-way ANOVA explored differences in smartphone use levels. Multinomial logistic regression, suitable for categorical nominal data without ordering and not requiring normality or linearity assumptions, was used to analyze the dependent variable (44). Analyses were performed using STATA 17.0, with significance set at p< 0.05.

Sample size adequacy was evaluated through *post-hoc* power analysis using G*Power 3.1 (45). For regression analysis with 22 predictors, n=1374, α=0.05, and a medium effect size (f²=0.15) based on previous smartphone research and Cohen’s guidelines, the achieved power exceeded 0.99. The overall sample-to-predictor ratio was 62.5 (1374/22), and the smallest outcome category (n=416, 30.28%) provided 18.9 events per variable, substantially exceeding the recommended minimum of 10 EPV for multinomial logistic regression (46, 47).

To assess potential multicollinearity, variance inflation factors (VIF) were calculated. All VIF values ranged from 1.04 to 3.74 (mean=1.47), well below the threshold of 5, indicating robust and reliable model estimates. These analyses collectively confirm that our sample size was more than adequate to detect meaningful effects while minimizing risks of Type II error and overfitting.

Model evaluation utilized multiple information criteria. While AIC emphasizes predictive accuracy, BIC favors parsimony. Given our exploratory research objectives and sample size, divergence between these metrics was anticipated and interpreted accordingly (48).

To investigate the moderating role of depression, we introduced interaction terms between depression status and other key variables, including weekly physical exercise and education level, into our multinomial logistic regression models. When a statistically significant interaction was detected, we performed *post-hoc* analyses to compute the predictive margins of excessive smartphone use for each subgroup. Subsequently, visualize these predictive margins, thereby illustrating the nature of the interaction effects.

## 3 Results

### 3.1 Characteristics of the respondents

**Table 1** presents descriptive statistics for the 1374 respondents, aged 12 to 89 years, with an average age of 47.89 years (SD = 13.89). Among them, 18.7% had hypertension, 1.53% had diabetes, and 32.39% had other chronic diseases; 61.28% had a normal BMI. The mean EQ-5D-5L score was 0.95 (SD = 0.12), indicating that most participants were close to full health. Regarding limitations in daily activities due to health, 81.3% were not limited. Additionally, 77.87% reported no insomnia in the past two weeks, 66.45% had minimal to no depressive symptoms (PHQ-9 score< 5), and 92.87% showed minimal anxiety symptoms (GAD-2 score< 3).

**Table 1:** Sample characteristics and univariate analysis of excessive smartphone use levels among 1374 respondents in Tibet.

Variables	All respondent (N = 1374)		Smartphone uses Levels						*χ^2^*/F	*Group difference (p-value)*
			Below average (n=457, 33.26)		Average (n=501, 36.46)		Excessive (n=416, 30.28)			
	N/mean	%/SD	N/mean	%/SD	N/mean	%/SD	N/mean	%/SD		
Biological factors:										
Age (age range 12~89)										
Teenager 12-19	14	1.02	1	7.14	6	42.86	7	50.00	39.070	<0.001***
Young adult 20-39	380	27.66	93	24.47	133	35.00	154	40.53		
Middle aged 40-65	834	60.70	305	36.57	305	36.57	224	26.86		
Old aged 66+	146	10.63	58	39.73	57	39.04	31	21.23		
Hypertension										
Yes	257	18.70	91	35.41	99	38.52	67	26.07	2.654	0.265
No	1117	81.30	366	32.77	402	35.99	349	31.24		
Diabetes										
Yes	21	1.53	3	14.29	8	38.10	10	47.62	4.443	0.108
No	1353	98.47	454	33.56	493	36.44	406	30.01		
Other chronic diseases										
Yes	445	32.39	155	34.83	162	36.40	128	28.76	0.986	0.611
No	929	67.61	302	32.51	339	36.49	288	31.00		
BMI										
Underweight	105	7.64	39	37.14	45	42.86	21	20.00	6.728	0.347
Normal weight	842	61.28	277	32.90	306	36.34	259	30.76		
Overweight	342	24.89	113	33.04	123	35.96	106	30.99		
Obese	85	6.19	28	32.94	27	31.76	30	35.29		
EQ-5D-5L descriptive system	0.95	0.12	0.95	0.12	0.95	0.12	0.96	0.10	0.800	0.451
Limitation of daily activities due to health										
Not limited	1117	81.30	369	33.03	397	35.54	351	31.42	7.702	0.103
Limited, but not severe	218	15.87	78	35.78	83	38.07	57	26.15		
Severely limited	39	2.84	10	25.64	21	53.85	8	20.51		
Insomnia symptoms										
Not at all	1070	77.87	343	32.06	392	36.64	335	31.31	8.427	0.208
Several days	214	15.57	75	35.05	78	36.45	61	28.50		
More than half the days	59	4.29	27	45.76	17	28.81	15	25.42		
Almost every day	31	2.26	12	38.71	14	45.16	5	16.13		
II. Psychological factors:										
Depression status										
PHQ-9 score<5	913	66.45	332	36.36	344	37.68	237	25.96	25.978	<0.001***
PHQ-9 score 5-9	254	18.49	70	27.56	84	33.07	100	39.37		
PHQ-9 score >=10	207	15.07	55	26.57	73	35.27	79	38.16		
Anxiety status										
GAD-2 score<3	1276	92.87	434	34.01	460	36.05	382	29.94	4.558	0.102
GAD-2 score >=3	98	7.13	23	23.47	41	41.84	34	34.69		
III. Social factors:										
Gender										
Man	677	49.27	193	28.51	243	35.89	241	35.60	21.664	<0.001***
Woman	697	50.73	264	37.88	258	37.02	175	25.11		
Regional location										
Shannan	622	45.27	257	41.32	244	39.23	121	19.45	96.115	<0.001***
Nyingchi	150	10.92	67	44.67	36	24.00	47	31.33		
Qamdo	602	43.81	133	22.09	221	36.71	248	41.20		
Urban-rural location										
Urban	435	31.66	151	34.71	166	38.16	118	27.13	2.993	0.224
Rural/Pastoral	939	68.34	306	32.59	335	35.68	298	31.74		
Education level										
Illiterate	619	45.05	202	32.63	229	37.00	188	30.37	19.339	0.001**
Primary school	545	39.67	204	37.43	199	36.51	142	26.06		
Junior high school or above	210	15.28	51	24.29	73	34.76	86	40.95		
Marital status										
Single	127	9.24	31	24.41	50	39.37	46	36.22	13.646	0.009**
Married	1150	83.70	380	33.04	422	36.70	348	30.26		
Separated/divorced/widowed	97	7.06	46	47.42	29	29.90	22	22.68		
Employment status										
Employed	1140	82.97	372	32.63	405	35.53	363	31.84	9.3272	0.053
Retired	16	1.16	8	50.00	5	31.25	3	18.75		
Unemployed	218	15.87	77	35.32	91	41.74	50	22.94		
Total household Income (RMB yuan)										
Less than 20,000	447	32.53	155	34.68	159	35.57	133	29.75	5.390	0.864
20,000-40,000	315	22.93	93	29.52	122	38.73	100	31.75		
40,000-60,000	231	16.81	81	35.06	86	37.23	64	27.71		
60,000-80,000	127	9.24	43	33.86	44	34.65	40	31.50		
80,000-100,000	82	5.97	24	29.27	28	34.15	30	36.59		
More than 100,000	172	12.52	61	35.47	62	36.05	49	28.49		
Drinking water type										
Tap water treated by centralized purification	94	6.84	19	20.21	22	23.40	53	56.38	37.057	<0.001***
Protected well water or spring water	1252	91.12	425	33.95	467	37.30	360	28.75		
Unprotected well water or spring water	28	2.04	13	46.43	12	42.86	3	10.71		
IV. Healthy lifestyle behavior factors:										
Smoking										
Smoker	243	17.69	70	28.81	87	35.80	86	35.39	4.347	0.114
Ex-smoker/ Non-smoker	1131	82.31	387	34.22	414	36.60	330	29.18		
Drinking										
Yes	221	16.08	64	28.96	84	38.01	73	33.03	2.298	0.317
No	1153	83.92	393	34.08	417	36.17	343	29.75		
Active health check-up										
Yes	894	65.07	309	34.56	335	37.47	250	27.96	6.541	0.038**
No	480	34.93	148	30.83	166	34.58	166	34.58		
Weekly physical exercise										
Never exercised	816	59.39	268	32.84	285	34.93	263	32.23	5.038	0.754
Less than once	37	2.69	13	35.14	15	40.54	9	24.32		
1–2 times	115	8.37	42	36.52	42	36.52	31	26.96		
3–5 times	134	9.75	44	32.84	55	41.04	35	26.12		
6 times or more	272	19.80	90	33.09	104	38.24	78	28.68		

$\chi^{2}$ : Chi-square test; F: One-way ANOVA test.

SD, standard deviation; *P< 0.1; **P< 0.05; ***P< 0.001.

EQ-5D-5L Scale reliability coefficient is 0.800.

PHQ-9 Scale reliability coefficient is 0.779; GAD-2 Scale reliability coefficient is 0.783.

The gender distribution was nearly even, with 49.27% male and 50.73% female. Participants were from Shannan (45.27%, n = 622), Nyingchi (10.92%, n = 150), and Qamdo (43.81%,n = 602) regions, with the sample distribution reflecting the population density of these regions (49). Most respondents (68.34%) lived in rural or pastoral areas, 45.05% were illiterate, 83.7% were married, and 82.97% were employed. Income levels showed that 32.53% had a total household income of less than RMB20,000. The majority (91.12%) used protected well or spring water, 82.31% were ex-smokers or non-smokers, 83.92% had not consumed alcohol in the past year, 65.07% had an active health check-up in the past year, and 59.39% never exercised.

### 3.2 Reliability and scores of variables

**Table 2** shows Cognitive salience had a mean score of 3.54 (SD = 1.84), with 38.72% excessive users, often thinking about their phones even when not in use. Behavioral salience averaged 3.51 (SD = 1.83), with 35.81% excessive users, showing habitual, purposeless phone use. Withdrawal symptoms had a mean of 3.53 (SD = 1.90), with 36.32% excessive users, indicating distress when separated from their phones. Overall, the total excessive smartphone use score averaged 10.57 (SD = 5.12), with 30.28% of respondents showing significant levels of excessive use.

**Table 2:** Excessive smartphone use scale scores and descriptive statistics for different items (N = 1374).

Item	Excessive use n (%)	Average use n (%)	Below average use n (%)	Mean ± SD
1. Cognitive Salience I often think about my smartphone when I am not using it.	532 (38.72)	301 (21.91)	541 (39.37)	3.54 ± 1.84
2. Behavioral salience I often use my smartphone for no particular reason.	492 (35.81)	336 (24.45)	546 (39.74)	3.51 ± 1.83
3. Withdrawal The thought of being without my smartphone makes me feel distressed.	499 (36.32)	284 (20.67)	591 (43.01)	3.53 ± 1.90
Total	416 (30.28)	501 (36.46)	457 (33.26)	10.57 ± 5.12

MPIQ Scale reliability coefficient is 0.909.

SD, Standard deviation.

### 3.3 Univariate analysis results

In **Table 1**, Chi-square tests (χ²) revealed significant differences in smartphone use levels across age groups (χ² = 37.771, p< 0.001). The 20–39 age group showed the highest rate of excessive smartphone use (40.53%), followed by the 40–65 age group (26.86%). Notably, we observed a surprisingly high rate of excessive smartphone use among those over 66 (21.23%), which contrasts with common assumptions about technology adoption in older populations. Hypertension, diabetes, other chronic diseases, BMI classification, EQ-5D-5L descriptive system, limitation of daily activities due to health, and insomnia symptoms were not significant.

For psychological factors, there was a significant association between PHQ-9 scores and smartphone use levels (χ² = 25.978, p < 0.001), with higher PHQ-9 scores significantly associated with smartphone use. No significant association was found between GAD-2 scores and smartphone use levels (χ²= 4.558, p = 0.102).

**Figure 1:**
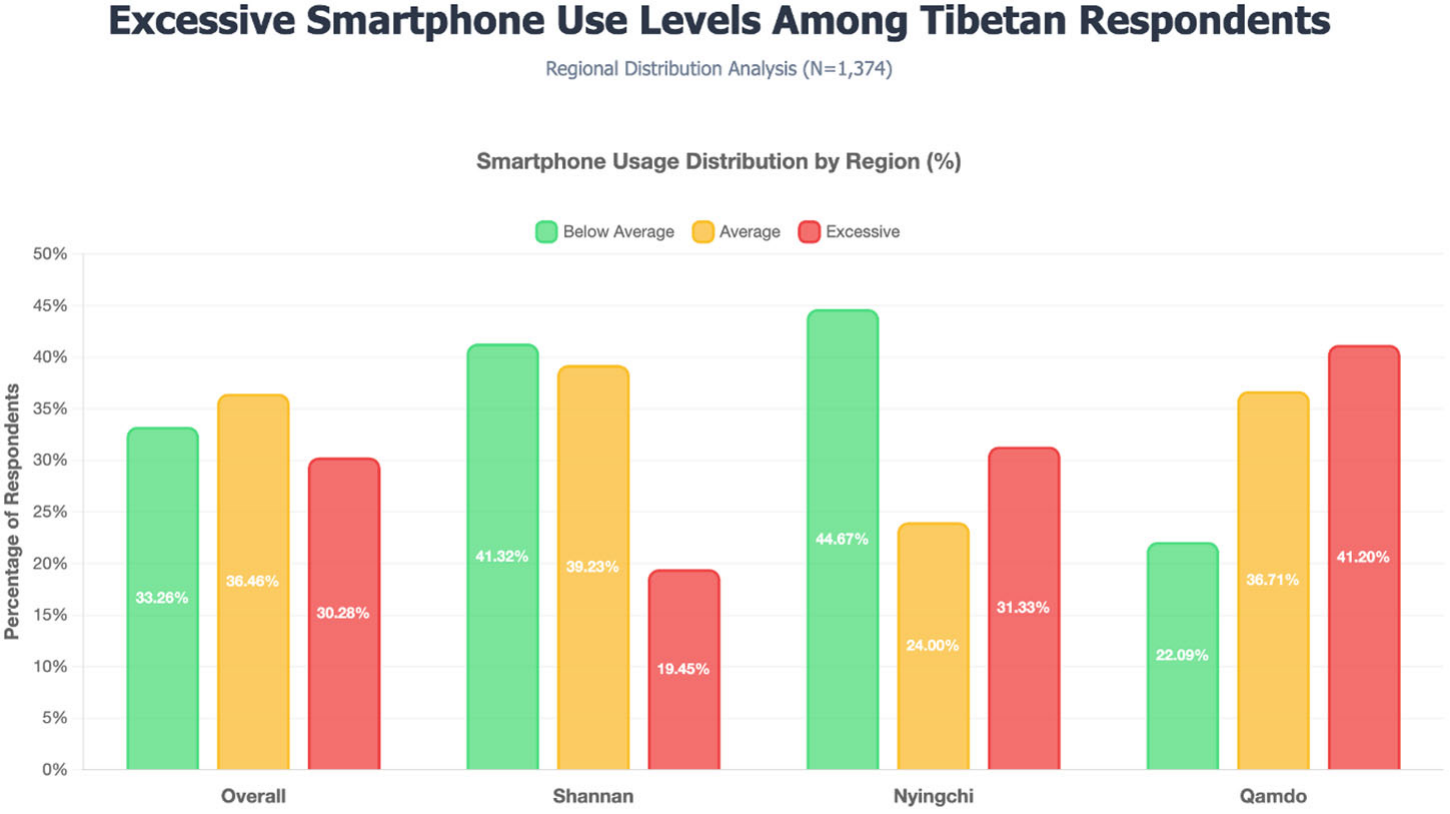
Excessive smartphone use levels among Tibetan respondents (N = 1374).

For social factors, a significant association was found between gender and smartphone use levels (χ²= 21.664, p < 0.001), with men having a higher proportion of excessive smartphone use compared to women. As shown in **Figure 1**, significant differences were observed in smartphone use levels across different regions (χ²= 96.115, p < 0.001), with respondents from Qamdo having the highest proportion of excessive use. A significant association was also found between the education level and smartphone use levels (χ²= 19.339, p = 0.001), indicating that higher education levels were associated with smartphone use, and a significant association between marital status and smartphone use (χ² = 13.646, p = 0.009), with single individuals showing the highest proportion of excessive use (36.22%) compared to married (30.26%) or separated/divorced/widowed (22.68%) individuals. Employment status was marginally associated with smartphone use levels (χ²= 9.327, p = 0.053). However, no significant association was found between residence area (urban/rural-pastoral) and smartphone use levels (χ²= 2.993, p = 0.224).

Turning to healthy lifestyle behavior factors, a significant association was found between an active health check-up and smartphone use levels (χ²= 6.541, p = 0.038), with those without an active health check-up more likely to be excessive smartphone users. Smoking status (p = 0.114), drinking status (p = 0.317), and weekly physical exercise (p = 0.754) were not significant across the groups.

### 3.4 Multinomial logistic regression analysis results

**Table 3** presents the multinomial logistic regression analysis, examining the associations between various attributes and excessive smartphone use levels. The null model’s log-likelihood value was -1505.547, which improved to -1366.226 after adding independent variables, indicating better model fit. The AIC decreased from 3015.093 to 2896.452, suggesting improved fit. However, the BIC increased from 3025.544 to 3324.942. This divergence between AIC and BIC is expected given their different penalty structures (48). With our sample of 1374 participants and multiple predictors, BIC’s stricter penalty for model complexity resulted in a higher value despite the improved log-likelihood. Following Raftery (50), we prioritized AIC for model selection as it better balances goodness-of-fit with model parsimony for our research objectives focusing on predictive accuracy rather than identifying the “true” model (50). The likelihood ratio test (χ² = 278.642, p< 0.001) confirms the full model significantly improves upon the null model.

**Table 3:** Multinomial logistic regression models examining associations between attributes and excessive smartphone use levels among 1374 respondents in Tibet.

Variables	Model 1: excessive vs below average use			Model 2: excessive vs average use		
	*β*	*P*	RRR (95CI)	*β*	*P*	RRR (95CI)
I. Biological factors:						
Age (Years)	-0.025	0.002**	0.975 (0.959,0.990)	-0.020	0.008**	0.980 (0.966,0.995)
Hypertension (ref=Yes)	-0.046	0.841	0.955 (0.611,1.494)	0.038	0.861	1.039 (0.678,1.591)
Diabetes (ref=Yes)	-1.574	0.034**	0.207 (0.048,0.888)	-0.636	0.234	0.529 (0.185,1.511)
Other Chronic Diseases(ref=Yes)	-0.231	0.186	0.794 (0.564,1.117)	-0.197	0.237	0.821 (0.592,1.138)
BMI classification (ref=underweight)						
Normal weight	0.332	0.291	1.394 (0.753,2.580)	0.426	0.158	1.531 (0.848,2.766)
Overweight	0.287	0.398	1.333 (0.684,2.597)	0.427	0.191	1.532 (0.808,2.905)
Obese	0.442	0.298	1.556 (0.676,3.582)	0.723	0.078	2.061 (0.923,4.600)
EQ-5D-5L descriptive system	0.154	0.853	1.166 (0.229,5.935)	-0.559	0.480	0.572 (0.121,2.697)
Limitation of daily activities due to health (ref=not limited)						
Limited, but not severe	-0.145	0.523	0.865 (0.554,1.351)	-0.242	0.270	0.785 (0.511,1.206)
Severely limited	-0.070	0.902	0.933 (0.307,2.831)	-0.945	0.059	0.389 (0.146,1.036)
Insomnia symptoms (ref=Not at all)						
Several days	0.021	0.926	1.021 (0.657,1.585)	0.011	0.961	1.011 (0.664,1.537)
More than half the days	-0.517	0.179	0.596 (0.280,1.268)	0.125	0.754	1.133 (0.519,2.477)
Nearly every day	-0.363	0.532	0.696 (0.223,2.169)	-0.682	0.226	0.506 (0.168,1.526)
II. Psychological factors:						
Depression status (ref=PHQ-9 score<5)						
PHQ-9 score 5-9	0.866	<0.001***	2.377 (1.599,3.533)	0.762	<0.001***	2.144 (1.483,3.099)
PHQ-9 score >=10	0.994	<0.001***	2.701 (1.697,4.300)	0.756	<0.001***	2.130 (1.402,3.235)
Anxiety status (ref= GAD-2 score<3)						
GAD-2 score >=3	0.185	0.555	1.203 (0.652,2.218)	-0.209	0.433	0.811 (0.481,1.369)
III. Social factors:						
Gender (ref=Man)						
Woman	-0.559	0.001**	0.572 (0.405,0.806)	-0.453	0.006**	0.636 (0.459,0.881)
Regional location (ref=Shannan)						
Nyingchi	0.428	0.085	1.534 (0.942,2.496)	0.960	<0.001***	2.612 (1.541,4.427)
Qamdo	1.433	<0.001***	4.191 (2.778,6.324)	0.658	0.001**	1.930 (1.325,2.811)
Urban-rural location (ref=urban)						
Rural-Pastoral	-0.137	0.472	0.872 (0.601,1.265)	0.009	0.959	1.010 (0.710,1.435)
Education Level (ref=illiterate)						
Primary school	0.062	0.727	1.064 (0.752,1.505)	0.055	0.744	1.057 (0.758,1.474)
Junior high school or above	0.586	0.024**	1.798 (1.082,2.986)	0.315	0.186	1.370 (0.859,2.184)
Marital status (ref=single)						
Married	-0.195	0.498	0.823 (0.468,1.446)	0.012	0.963	1.012 (0.612,1.672)
Separated/divorced/widowed	-0.400	0.331	0.670 (0.299,1.502)	0.317	0.430	1.373 (0.625,3.021)
Employment status (ref=employed)						
Retired	-0.939	0.239	0.391 (0.082,1.864)	-0.862	0.294	0.422 (0.084,2.112)
Unemployed	0.009	0.972	1.009 (0.605,1.684)	-0.284	0.243	0.753 (0.468,1.213)
Total household income (ref=less than 20,000)						
20,000-40,000	0.346	0.097	1.413 (0.939,2.125)	0.040	0.837	1.041 (0.711,1.523)
40,000-60,000	0.268	0.254	1.307 (0.825,2.071)	-0.002	0.992	0.998 (0.643,1.547)
60,000-80,000	0.157	0.582	1.170 (0.669,2.046)	-0.026	0.925	0.975(0.570,1.665)
80,000-100,000	0.738	0.030**	2.091 (1.076,4.062)	0.354	0.260	1.425 (0.769,2.639)
More than 100,000	0.260	0.323	1.297 (0.774,2.172)	0.020	0.939	1.020 (0.619,1.680)
Drinking water type (ref=tap water treated by centralized purification)						
Protected well water or spring water	-0.902	0.007**	0.406 (0.212,0.777)	-1.071	<0.001***	0.343 (0.190,0.616)
Unprotected well water or spring water	-3.230	<0.001***	0.040 (0.009,0.172)	-2.792	<0.001***	0.061 (0.146,0.258)
IV. Healthy lifestyle behavior factors:						
Smoking (ref=smoker)						
Ex-smoker/Non-smoker	-0.149	0.484	0.861 (0.567,1.309)	-0.061	0.758	0.941 (0.639,1.387)
Drinking (ref=yes)						
No	-0.319	0.131	0.727 (0.481,1.099)	0.002	0.991	1.002 (0.682,1.474)
Active health check-up (ref=yes)						
No	0.418	0.794	1.043 (0.762,1.426)	0.116	0.443	1.123 (0.835,1.512)
Weekly physical exercise (ref=never)						
Less than once	-0.399	0.402	0.671 (0.264,1.704)	-0.398	0.383	0.672(0.275,1.643)
1–2 times	-0.736	0.011**	0.479 (0.271,0.847)	-0.539	0.054	0.584(0.338,1.008)
3–5 times	-1.068	<0.001***	0.344 (0.197,0.599)	-0.833	0.002**	0.435(0.260,0.727)
6 times or more	-0.355	0.088	0.701 (0.466,1.054)	-0.356	0.066	0.700(0.479,1.024)

*P< 0.1; **P< 0.05; ***P< 0.001.

β, regression coefficient.

RRR, relative risk ratio.

CI, confidence interval.

For biological factors, age was significantly negatively associated with excessive smartphone use in both models. Each additional year of age decreased the relative risk ratio (RRR) of excessive smartphone use by 2.5% in Model 1 (RRR = 0.975, *p* = 0.002) and by 2.0% in Model 2 (RRR = 0.980, *p* = 0.008). In Model 1, having diabetes significantly reduced the RRR of excessive smartphone use (RRR = 0.207, *p* = 0.034), though this was not significant in Model 2. No other biological variables showed significant associations with excessive smartphone use in either model.

For psychological factors, higher PHQ-9 scores, indicating greater depression, were significantly associated with a higher likelihood of excessive smartphone use in both models. For PHQ-9 scores of 5-9, the RRR was 2.377 (p < 0.001) in Model 1 and 2.144 (p < 0.001) in Model 2. For scores of 10 or more, the RRR was 2.701 (p < 0.001) in Model 1 and 2.130 (p < 0.001) in Model 2. Anxiety did not significantly associate with excessive smartphone use in either model.

For the social factors, females had a lower likelihood of excessive smartphone use compared to males in both models (Model 1: RRR = 0.572, *p* = 0.001; Model 2: RRR = 0.636, *p* = 0.006). Regional location also showed significant effects: living in Qamdo was associated with a higher RRR of excessive smartphone use compared to living in Shannan in both models (Model 1: RRR = 4.191, *p* < 0.001; Model 2: RRR = 1.930, *p* = 0.001). Living in Nyingchi was associated with a higher likelihood of excessive smartphone use in Model 2 (RRR = 2.612, *p* < 0.001). Having a junior high school education or above was associated with a higher RRR of excessive smartphone use compared to being illiterate in Model 1 (RRR = 1.798, *p* = 0.024) and compared to a total household income of less than RMB20,000, an income of RMB80,000-100,000 was associated with a higher RRR of excessive smartphone use in Model 1 (RRR = 2.091, *p* = 0.030). Compared to using tap water treated by centralized purification, using protected well water or spring water (Model 1: RRR = 0.406, *p* = 0.007; Model 2: RRR = 0.343, *p* < 0.001) and unprotected well water or spring water (Model 1: RRR = 0.040, *p<* 0.001; Model 2: RRR = 0.061, *p* < 0.001) were associated with a lower RRR of excessive smartphone use.

For healthy lifestyle behavior factors, regular physical exercise was significantly negatively associated with excessive smartphone use. Exercising 3–5 times a week was particularly effective in reducing the likelihood of excessive smartphone use (Model 1: RRR = 0.344, p < 0.001; Model 2: RRR = 0.260, p = 0.002). Smoking, drinking, and active health check-ups were not significantly associated with excessive smartphone use in either model.

### 3.5 Interaction effects of depression

**Figure 2:**
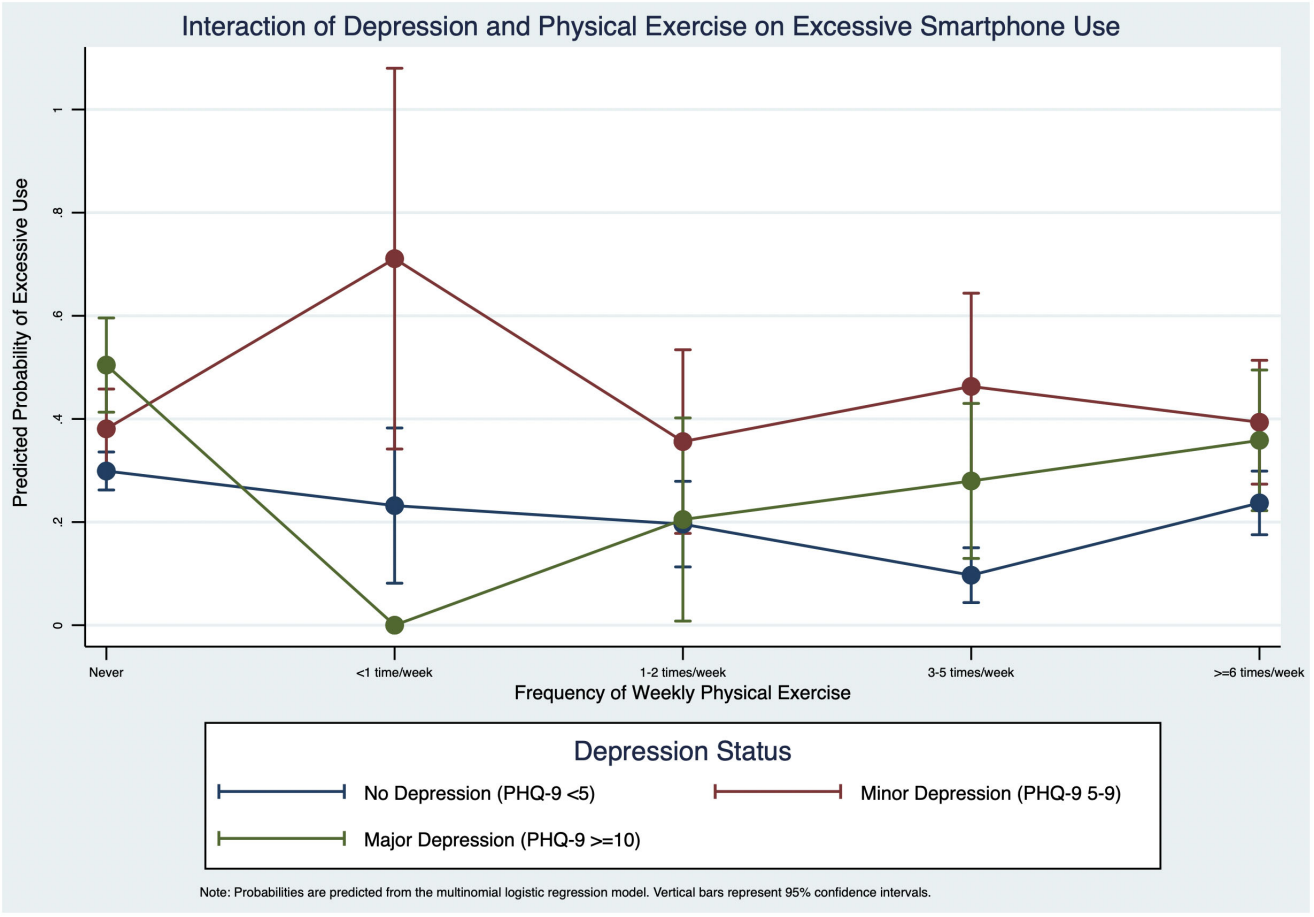
Interaction of depression and physical exercise on excessive smartphone use.

To visualize the significant interaction between depression and physical exercise, we plotted the predictive margins of excessive smartphone use (**Figure 2**). The results revealed a strong interaction, where the effect of physical exercise was conditional on depression status. For individuals with minor depression, physical exercise appeared to be a protective factor; the probability of excessive use peaked at 71.1% for those exercising less than once a week, and steadily declined as exercise frequency increased. In stark contrast, for those with major depression, physical exercise acted as a risk factor. Their probability of excessive use was nearly zero when exercising less than once a week but rose consistently to 35.9% for those exercising six or more times per week. For the non-depressed group, the probability of excessive use remained low, exhibiting a U-shaped pattern that was lowest (9.7%) for those exercising 3–5 times per week.

**Figure 3:**
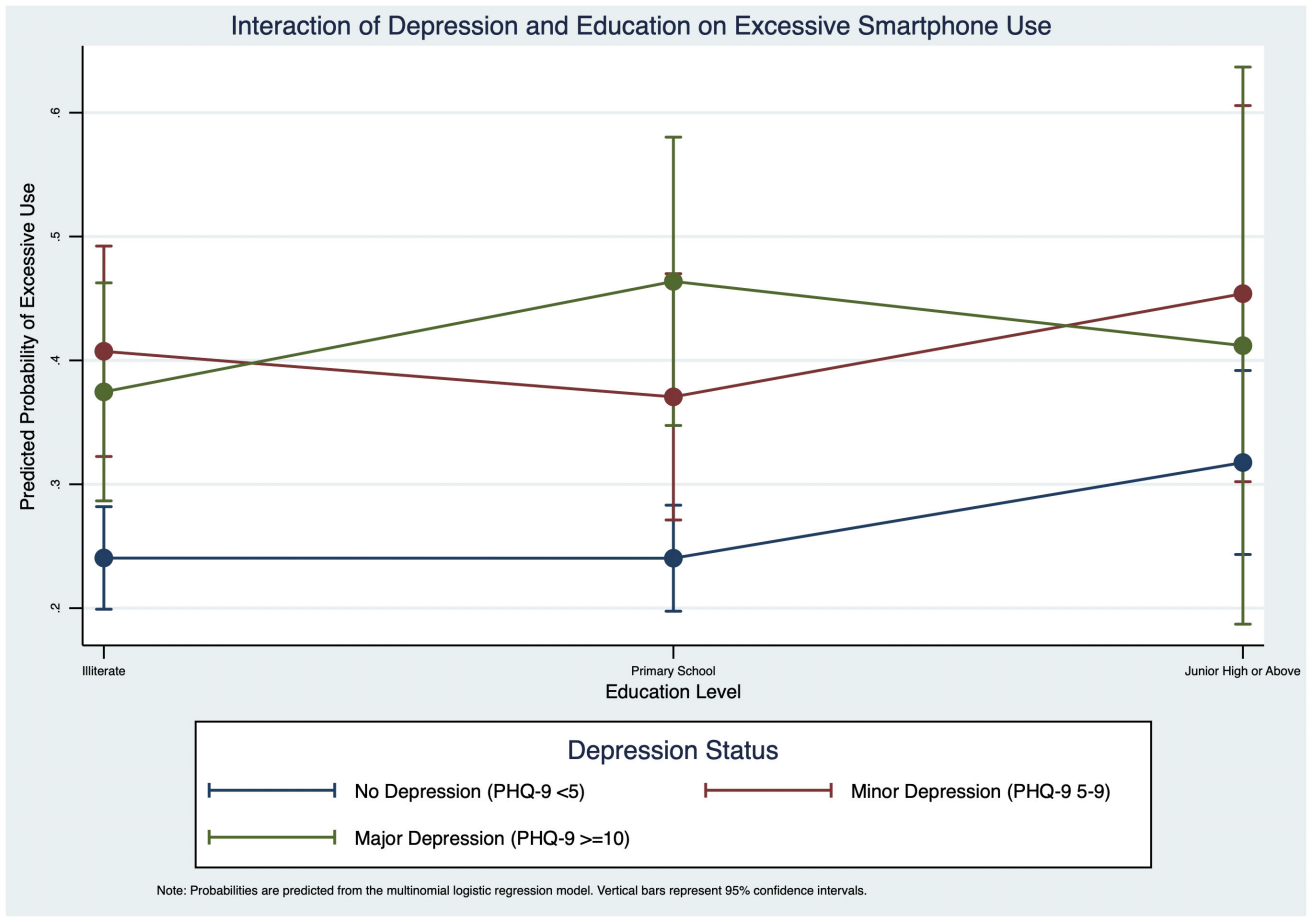
Interaction of depression and education level on excessive smartphone use.

Furthermore, we explored the interaction between depression and education level (**Figure 3**). This analysis also showed a significant moderating effect. For the non-depressed group, higher education was associated with a greater probability of excessive use, increasing from 24.1% for the illiterate group to 31.8% for those with junior high education or above. This positive trend was not observed in the depressed groups. For the minor depression group, the probability of excessive use remained consistently high (between 37.1% and 45.4%) across all education levels. For the major depression group, the relationship was non-linear, with the probability peaking at 46.4% for those with a primary school education.

## 4 Discussion

### 4.1 Main findings

Among 1374 respondents from three Tibetan regions in **Table 2**, 33.26% were below average, 36.46% average, and 30.28% were excessive smartphone users. There was surprisingly excessive smartphone use by those 66+ (21.23%) and 40–65 middle age (26.86%) groups, although the young adult 20–39 age group (40.53%) had the highest excessive smartphone use. Excessive smartphone use was higher in Qamdo and Nyingchi compared to Shannan. High depression scores and higher education levels were linked to excessive smartphone use, while females and those engaging in regular exercise were less likely to exhibit excessive use. The type of drinking water was significant, with those using well or spring water less likely to be excessive smartphone users than those using purified tap water, which reflected regional and socioeconomic factors. Higher household income was associated with increased smartphone use. Other factors showed no significant associations.

### 4.2 Comparison with existing literature

Previous research has predominantly focused on youth smartphone use, exploring various behavioral and psychological aspects (10, 51, 52). In contrast, this study examines smartphone addiction across the general population, moving beyond the narrow emphasis on adolescents and university students. Our results reflect recent global trends reported by Kumar (13), who found that smartphones are now used by 85% of the world’s population, with growing adoption among all age groups (13). The high rate of excessive use among younger adults (aged 20–39) in our sample is consistent with international patterns—for example, 34.8% of young adults in Turkey met criteria for smartphone addiction, and 77.3% used their devices for more than four hours daily (53), mirroring the behaviors we observed.

Notably, our study reveals that problematic smartphone use is also prevalent within the 40–65 age group. Applying a uniform addiction framework to this population is inadequate without accounting for their distinct life circumstances. For this “sandwich generation,” high smartphone usage is often motivated by instrumental needs and caregiving responsibilities—a dimension especially relevant in Tibet, where maintaining family connections despite outward migration is highly valued. Here, smartphones serve as essential tools for fulfilling these duties. However, a crucial limitation of the MPIQ scale is its focus on behavioral symptoms of addiction (e.g., loss of control, withdrawal) rather than underlying motivations. This is significant because we propose that even purpose-driven smartphone use can transition into problematic behavior. In highly connected cultural settings, the line between purposeful use and compulsive distraction is often blurred; checking a family message can lead to prolonged, uncontrolled scrolling. Thus, the same devices used for family obligations may facilitate behavioral dependency, with unintended consequences such as anxiety and sleep disruption. Although the initial drivers of use differ from those of younger users, middle-aged adults remain vulnerable to negative behavioral outcomes. This highlights a key constraint of our methodological approach: the MPIQ does not distinguish between motivation and behavior, indicating that a simplistic addiction framework is insufficient. Future studies should adopt mixed-methods designs to better unravel these complex interactions.

We found a strong association between higher PHQ-9 scores and increased excessive smartphone use, underscoring the importance of implementing mental health interventions targeting depression to mitigate smartphone overuse. These results are further supported by a large body of international research, which has consistently linked excessive smartphone use to negative mental health outcomes among young people (54, 55). Our study did not find a significant link between anxiety and excessive smartphone use, a finding that contrasts with some previous research. This discrepancy may be explained by key differences in sample populations, measurement tools, and cultural context. Much of the existing literature has focused on adolescents and university students (54, 55), whereas our study includes a general population of all ages, whose life stressors and anxiety triggers may be fundamentally different. Furthermore, the relationship may be contingent on the specific type of anxiety. We used the GAD-2 to screen for generalized anxiety, but other research suggests problematic smartphone use is more directly linked to social anxiety, a dimension not captured by our tool. (56). It is also possible that the unique cultural practices in Tibet, such as Tibetan Buddhism and prayer rituals, act as a buffer against anxiety, thus weakening any potential association with smartphone overuse. Taken together, our finding suggests the link between anxiety and excessive smartphone use is not universal but is complex and context-dependent. Future research should therefore employ more specific measurement tools to investigate this relationship across diverse demographic and cultural groups.

A particularly complex picture emerged regarding the role of chronic health conditions. In our initial univariate analysis, we found no statistically significant association between the presence of chronic diseases—such as hypertension or diabetes—and the levels of excessive smartphone use. However, after controlling for a wide range of demographic and socioeconomic factors, a significant negative association emerged specifically for a diabetes diagnosis, indicating a lower likelihood of excessive use compared to below-average use. We believe this finding should be interpreted with extreme caution. Our primary explanation is that this is likely a statistical artifact due to the extremely small sample size of participants with diabetes (n=21, 1.53%). The instability of this estimate is underscored by its inconsistency across the model’s comparisons—it was significant only when comparing the ‘excessive use’ group to the ‘below-average use’ group, but not against the ‘average use’ group. While we attribute this finding primarily to statistical limitations, it is plausible that the underlying mechanism connects back to the smartphone’s role. Managing a serious illness like diabetes may encourage more purposeful, tool-based smartphone engagement rather than the aimless, compulsive behavior captured by our scale (57). Ultimately, our study suggests the relationship between chronic illness and smartphone use is not straightforward and requires future research with larger sample sizes and more nuanced measurement tools to disentangle these complex interactions.

Our study found that individuals with higher education levels were more likely to report excessive smartphone use than the illiterate group. This seemingly paradoxical finding can be understood through the lens of the “digital divide”. First, higher education is strongly linked to higher rates of smartphone ownership and access. Global research consistently shows that individuals with higher level education more likely to be smartphone users (58). Second, education level is a primary factor in digital proficiency, as more educated individuals can more easily overcome technical complexities and are more likely to integrate information and communication technologies (ICT) into their work and daily lives (59). This combination of greater access and higher proficiency leads to a more intensive and multifaceted use of smartphones.

Further, educated people are more likely to use smartphones for multiple purposes, including study, work, social interactions and entertainment, further contributing to excessive smartphone use risks (60). Regional location also played a significant role in Tibetan excessive smartphone use. Compared to Shannan, living in Qamdo and Nyingchi was associated with higher excessive smartphone use levels. While zhandui et al. documented different population growth patterns across these regions (49), the specific mechanisms linking regional characteristics to smartphone use patterns remain unclear and warrant further investigation with more comprehensive regional socioeconomic data. We found no significant difference in excessive smartphone use between urban and rural-pastoral areas, aligning with studies of Chinese students showing similar results (61). Tibet’s unique socio-cultural and geographical context, with relatively low population density even in cities and widespread smartphone ownership, contribute to this finding.

Higher household income emerged as a risk factor for excessive smartphone use, likely due to greater access to digital devices and more leisure time among affluent individuals. This finding aligns with previous studies of college students and young adults, in which high household income was also identified as a significant predictor of problematic use (62). The underlying mechanisms appear multifaceted: financial resources facilitate device ownership and connectivity, while certain high-income lifestyles may offer more unstructured time, thereby increasing opportunities for engagement with smartphones. These insights highlight the importance of developing targeted interventions tailored to high-income groups, where excessive use may be driven by distinct socioeconomic and behavioral factors.

The type of drinking water, an indicator of housing conditions and socioeconomic status (19), was linked to smartphone use. Households using well or spring water, common in poorer rural areas, were less likely to engage in excessive smartphone use than those with purified tap water. Rural households, often inhabited by Tibetan herders, maintain a traditional lifestyle focused on community-based management of resources and outdoor activities like herding (63). This lifestyle fostered strong social connections through face-to-face interactions and collaborative daily activities, contributing to lower smartphone use. Our study also explored the role of marital status. While the initial univariate analysis indicated a significant difference, with single individuals reporting the highest rates of excessive use, it is crucial to note that this effect did not remain significant in our final multivariate regression model. This suggests that the influence of marital status is likely mediated or confounded by other, more powerful predictors in the model, such as age and depression. For instance, single individuals in our sample were, on average, younger, and age was one of the strongest predictors of smartphone use.

Regular physical exercise emerged as a significant protective factor against excessive smartphone use. Our finding that exercising 3–5 times per week substantially reduced the risk is corroborated by international evidence. For instance, a study of Turkish university students demonstrated that higher physical activity levels were associated with significantly lower smartphone addiction scores (64). This inverse relationship is further supported by a systematic review confirming that exercise interventions effectively reduce problematic smartphone use in both the short and long term (9) These consistent findings suggest that physical activity not only counteracts sedentary behaviors but may also serve as a compelling alternative to screen time, thereby reducing dependency on smartphones. Consequently, promoting an active lifestyle should be a cornerstone of public health strategies aimed at mitigating smartphone overuse. This can be achieved by integrating physical activity into daily routines through expanded access to public sports facilities, community wellness programs, workplace initiatives, and school curricula.

Consistent with prior research, we found no significant link between excessive smartphone use and smoking or alcohol consumption. This finding aligns with several international studies. One study found no association between lifestyle habits such as smoking and alcohol drinking with smartphone addiction among Lebanese university students. Similarly several study also reported that smoking and alcohol consumption were not significantly associated with smartphone addiction among medical students (65, 66). The absence of association in our study may be particularly influenced by the unique characteristics of our Tibetan sample. The low prevalence of smoking (17.69%) and alcohol consumption (16.08%) in our participants is limited variance in substance use behaviors within our culturally homogeneous sample may have reduced our statistical power to detect potential associations that might exist in populations with higher and more heterogeneous substance use patterns. Future studies examining these relationships in populations with greater variability in smoking and drinking behaviors may reveal different patterns of association.

Recent international data from 2025 shows a worldwide increase in smartphone use, with significant regional variations. For instance, while countries like the Philippines and Thailand report the highest average daily screen time, China has the highest number of total users and scores highest on measures of problematic smartphone addiction (13). This positions China, and by extension its diverse regions like Tibet, as a critical area for studying the drivers and consequences of high smartphone engagement. Research suggests that countries with high levels of problematic use often exhibit strong collectivism and “cultural tightness,” where formal social and family obligations create a powerful incentive for constant digital contact (67, 68). Our study deepens this perspective. We argue that in the Tibetan context, collectivism fundamentally reshapes the function of the smartphone, which may explain its high prevalence. Far from being a tool for individualistic escape, the smartphone becomes a vital conduit for maintaining intergenerational relationships and sustaining community ties, especially amidst labor migration. In this light, high usage is not merely a symptom of addiction but a modern expression of a core cultural value. This imperative to maintain social cohesion could be a key driver behind the high engagement rates we observed across all age groups. Furthermore, the sociocultural context may also shape the lived experience of high usage. While not directly measured, the pervasive influence of Tibetan Buddhism, with its emphasis on mindfulness and impermanence, could act as a psychological buffer. It is plausible that these cultural practices equip individuals with cognitive tools to mitigate the anxiety-provoking aspects of hyper-connectivity, even if they do not reduce usage itself. This might partially explain why we found a strong link between smartphone use and depression, but not with generalized anxiety. This suggests that culture does not just influence how much people use their phones, but also how they emotionally process that use.

### 4.3 Public health and policy implications

Our findings provide several actionable insights for developing targeted, culturally resonant public health policies to address excessive smartphone use in the Tibetan context. A top-down, one-size-fits-all approach is unlikely to succeed; instead, interventions must be woven into the existing social and cultural fabric.

Integrate Mental Health Support with Community Trust: The strong link between depression and excessive smartphone use highlights an urgent need for mental health support. However, to destigmatize these issues and ensure uptake, clinical screening must be integrated with community-led efforts. This involves collaborating with local community leaders, who are trusted voices in Tibetan society. These leaders can be trained to recognize signs of distress and can help frame mental well-being not as a clinical problem, but as a component of a balanced and healthy life, thereby encouraging individuals to access local health services without fear of judgment.

Promote Physical Activity through Cultural Practice: Given that regular physical exercise is a powerful protective factor, policies should focus on embedding activity within daily life. Beyond simply building facilities, this means actively organizing community events centered on traditional cultural practices. For example, public health initiatives could support regular gatherings for traditional Tibetan group dances (e.g., Guozhuang) or sports, timing them with local festivals to maximize participation. Such activities are not only physical but also reinforce strong social bonds, directly countering the digital isolation that can fuel smartphone overuse.

Deliver Tailored Interventions through Trusted Channels: Different demographics require different messages, and these messages must be delivered effectively. Rather than relying on generic media campaigns, awareness efforts should leverage traditional community gatherings and local networks. For instance, discussions about the risks of an “always-on” culture for high-income groups, or the impact of gaming on family responsibilities for men, can be initiated by respected elders or community figures during these gatherings. This approach ensures the message is received from a credible source and is discussed within a supportive community context, making it far more resonant and effective than impersonal public service announcements.

The significant regional variations we observed also underscore that resource allocation and intervention efforts should be prioritized for regions like Qamdo and Nyingchi, with programs tailored to their unique socioeconomic and cultural landscapes.

### 4.4 Strengths and limitations

When interpreting our results, the following limitations should be considered: first, the cross-sectional design of this study limits its ability to establish causal relationships between variables related to smartphone addiction in Tibet. Second, while the study addresses the gap in research on smartphone addiction in Tibet, the sample may not be sufficiently representative to generalize the findings to the entire nation. Third, despite quality control measures, reliance on self-reported data introduces potential response bias and subjectivity. Fourth, our adapted three-item version of the Mobile Phone Involvement Questionnaire (MPIQ), while practical and capturing core addiction components (salience and withdrawal), has inherent limitations. The scale primarily measures behavioral patterns without distinguishing underlying motivations for use. This is particularly relevant for middle-aged adults (40–65 years), where high usage often reflects instrumental necessity (e.g., work coordination, family caregiving) rather than compulsive behavior. The MPIQ might classify such functionally essential usage as ‘excessive,’ potentially masking nuanced realities of smartphone use in this population.

Fifth, our study did not collect several potentially important variables related to usage patterns and device characteristics. We did not measure the duration of smartphone ownership, which prevented us from distinguishing between the effects of long-term versus short-term use. We also did not record objective usage time (e.g., daily screen hours) or the screen size of the devices. These factors could influence usage behavior and health outcomes. Sixth, while our inclusion criteria excluded individuals with observable cognitive impairments, we did not use a formal screening tool (e.g., MMSE) to systematically assess the cognitive function of elderly participants. Finally, as with any cross-sectional study, our findings are susceptible to potential confounding from underlying variables. A specific concern was that observed associations (particularly with age) could be confounded by participants’ chronic health status or the motivational factors mentioned above. While our sensitivity analysis (**Appendix Table 1**) confirmed the robustness of main findings in a healthy subsample (n=775), we acknowledge that unmeasured health factors and the motivation-usage distinction may contribute to residual confounding.

Future research should address these methodological gaps by: incorporating longitudinal designs, using mixed-methods approaches (combining quantitative scales with qualitative interviews) to better distinguish between necessary and compulsive usage, collecting objective usage data, and obtaining more detailed participant information about device characteristics and usage contexts.

Despite these limitations, our study possesses several significant strengths that provide a critical contribution to the literature on behavioral addictions. First and most importantly, our research moves beyond the conventional focus on youth and university students, investigating smartphone use across the full adult lifespan in a general population. By including a large and diverse sample of teenagers, middle-aged adults, and the elderly, our findings offer a rare and more holistic understanding of how smartphone engagement varies across different life stages, a perspective conspicuously absent in most existing studies.

Second, our study is situated in the unique and under-researched sociocultural context of Tibet. By applying the biopsychosocial model to a non-Western, ethnic minority population, we not only test the model’s cross-cultural applicability but also illuminate how distinct cultural values—such as collectivism and local traditions—may shape the drivers and manifestations of problematic smartphone use. This provides a crucial counter-narrative to research predominantly centered on urban, Han-majority populations.

Finally, the study’s robust methodological design, including a large, systematically randomized sample (n=1374) and rigorous statistical analysis, ensures the reliability of our findings. By identifying key risk factors (e.g., depression, male gender, higher income) and protective factors (e.g., physical exercise) within this specific demographic, our research successfully lays an evidence-based foundation for developing culturally-tailored public health interventions. While further longitudinal research is needed, these findings pave the way for new preventive and regulatory policies designed for a population at the intersection of tradition and rapid modernization.

## 5 Conclusions

This study provides a comprehensive analysis of the factors associated with excessive smartphone use across the general population in Tibet, moving beyond the typical focus on youth. Our findings reveal a complex interplay of biopsychosocial and behavioral factors. Younger age, male gender, higher depression scores, higher education levels, and higher income were identified as significant risk factors, while regular physical exercise emerged as a strong protective factor.

Notably, the drivers of excessive use are highly context-dependent. For instance, middle-aged adults face unique pressures related to work-life entanglement that shape their usage patterns. Furthermore, unique socioeconomic indicators, such as the type of drinking water, were also significantly associated with smartphone use, reflecting the deep influence of local living conditions and traditional lifestyles.

This study underscores that excessive smartphone use in this unique cultural context is not merely a youth issue but a widespread phenomenon affecting all demographics, driven by a combination of psychological distress, socioeconomic dynamics, and modern lifestyle pressures. Therefore, effective public health interventions must be holistic and tailored, rather than monolithic. Strategies should prioritize the integration of mental health support, vigorously promote physical activity, and be designed with sensitivity to the specific needs of different demographic groups and regions, all while leveraging Tibet’s strong community and cultural assets. These findings lay the groundwork for evidence-based policies aimed at fostering a healthier human-technology balance in a rapidly modernizing society.

## Data Availability

The raw data supporting the conclusions of this article will be made available by the authors, without undue reservation.

## Appendix Table 1

**Table:** Multinomial Logistic Regression on Healthy Subgroup (n=775).

Variables	Excessive use vs below average use	
I. Biological factors:	*β*	*P*
Age (Years)	-0.039	0.001***
BMI classification (ref=underweight)		
Normal weight	0.603	0.205
Overweight	0.512	0.316
Obese	0.536	0.401
EQ-5D-5L descriptive system	-4.519	0.025**
Limitation of daily activities due to health (ref=not limited)		
Limited, but not severe	-5.335	0.208
Severely limited	-4.401	0.289
Insomnia symptoms (ref=not at all)		
Several days	0.507	0.185
More than half the days	-1.659	0.077
Nearly every day	-1.185	0.853
II. Psychological factors:		
Depression status (ref=PHQ-9 score<5)		
PHQ-9 score 5-9	0.903	0.002**
PHQ-9 score >=10	1.410	<0.001***
Anxiety status (ref= GAD-2 score<3)		
GAD-2 score >=3	0.554	0.312
III. Social factors:		
Gender (ref=man)		
Woman	-0.668	0.009**
Regional location (ref=Shannan)		
Nyingchi	0.404	0.255
Qamdo	1.968	<0.001***
Urban-rural location (ref=urban)		
Rural-Pastoral	-0.287	0.281
Education level (ref=illiterate)		
Primary school	0.080	0.757
Junior high school or above	0.863	0.013**
Marital status (ref=single)		
Married	-0.412	0.312
Separated/divorced/widowed	-0.331	0.619
Employment status (ref=employed)		
Retired	-9.379	0.991
Unemployed	-0.588	0.268
Total household income (ref=less than 20,000)		
20,000-40,000	0.461	0.123
40,000-60,000	0.150	0.689
60,000-80,000	0.364	0.349
80,000-100,000	0.327	0.482
More than 100,000	0.381	0.288
Drinking water type (ref=tap water treated by centralized purification)		
Protected well water or spring water	-0.414	0.388
Unprotected well water or spring water	-3.128	<0.001***
IV. Healthy lifestyle behavior factors:		
Smoking (ref=smoker)		
Ex-smoker/Non-smoker	-0.022	0.939
Drinking (ref=yes)		
No	-0.919	0.111
Active health check-up (ref=yes)		
No	-0.211	0.338
Weekly physical exercise (ref=never)		
Less than once	-0.768	0.302
1–2 times	-1.235	0.005**
3–5 times	-1.220	0.002**
6 times or more	-0.416	0.164

**P<* 0.1; ***P<* 0.05; ****P<* 0.001. β, regression coefficient; RRR, relative risk ratio; CI, confidence interval.

## Appendix Table 2

**Table:** Distribution of chronic conditions across age groups (N = 1,374).

Condition	Age group	Yes	No (n, %)
Hypertension	Teenager 12-19	0 (0.0%)	14 (100.0%)
	Young adult 20-39	4 (1.1%)	376 (98.9%)
	Middle aged 40-65	168 (20.1%)	666 (79.9%)
	Old aged 66+	85 (58.2%)	61 (41.8%)
	**Total**	**257 (18.7%)**	**1117 (81.3%)**
Diabetes	Teenager 12-19	0 (0.0%)	14 (100.0%)
	Young adult 20-39	2 (0.5%)	378 (99.5%)
	Middle aged 40-65	15 (1.8%)	819 (98.2%)
	Old aged 66+	4 (2.7%)	142 (97.3%)
	**Total**	**21 (1.5%)**	**1353 (98.5%)**
Other Chronic Diseases	Teenager 12-19	1 (7.1%)	13 (92.9%)
	Young adult 20-39	74 (19.5%)	306 (80.5%)
	Middle aged 40-65	297 (35.6%)	537 (64.4%)
	Old aged 66+	73 (50.0%)	73 (50.0%)
	**Total**	**445 (32.4%)**	**929 (67.6%)**

Bold values indicate the total prevalence (n, %) of each chronic condition across the entire sample (N = 1,374).
